# Optimal diapause strategies of a grasshopper, Melanoplus sanguinipes

**DOI:** 10.1673/1536-2442(2006)6[1:ODSOAG]2.0.CO;2

**Published:** 2006-04-07

**Authors:** Dennis Fielding

**Affiliations:** USDA Agricultural Research Service, P. O. Box 750102, Fairbanks, Alaska 99775

**Keywords:** life history theory, Orthoptera:Acrididae, life cycle, plasticity, seasonality

## Abstract

Previous analyses of diapause in insects have most often focused on the timing of the switch from non-diapausing to diapausing offspring in bivoltine populations and have assumed that diapause is irreversible or that the insect cannot survive winter if not in diapause. Many insects exhibit more flexibility in their life cycles, such as the age at which diapause begins, and facultative diapause, that may influence the evolution of different diapause strategies in different environments. The grasshopper Melanoplus sanguinipes F. (Orthoptera: Acrididae), has a very wide geographic range over which diapause characteristics vary greatly. Embryonic diapause in this species may be under maternal control, may be obligate or facultative (i.e., may be averted by cold temperature treatment of pre-diapause embryos), and embryos may enter diapause at different ages. Diapause traits were examined in two populations of M. sanguinipes from very different environments. In the population from a temperate climate (Idaho, USA), diapause was facultative, i.e., pre-diapause embryos averted diapause when held at 5° C for 90 days at all ages tested (7 days and older). The Idaho embryos entered diapause in late stage of development if held at 22° C for 30 days or more. In populations from subarctic Alaska, USA, embryos also entered diapause in a late stage of development, but diapause was obligate and could not be averted by chilling in the pre-diapause stages. Simulated evolution of these traits over a wide range of season-lengths showed that late stage diapause is an essential trait in very short season environments, resulting in early hatching, and a semivoltine life-cycle. Facultative diapause enabled bivoltinism to be a viable strategy in shorter seasons than when diapause was obligate. At transitions from semivoltine to univoltine, and from univoltine to bivoltine life cycles, populations with obligate diapause adopted a strategy of no diapause (via maternal effects) to enable univoltine life cycles.

## Introduction

Diapause is a key life history trait that ensures that an insect's life cycle is synchronized with seasonal changes of environment (Tauber et al. 1986). Numerous studies with various species of grasshoppers have demonstrated a wide variety of diapause traits that play an adaptive role in their life cycles (Wardhaugh 1986; Cherrill & Begon 1991; Groeters 1994; Tanaka 1994; Gehrken & Doumbia 1996; Hunter et al. 2001). Most species of acridids, including Melanoplus sanguinipes F. (Orthoptera: Acrididae), overwinter as eggs in the temperate grasslands of North America (Otte 1981, 1984; Pfadt 1994). Embryonic diapause in these species halts morphological development to ensure that hatching does not occur too late in the season for the individual to complete development and reproduce before the onset of winter. The extremely broad geographic range of M. sanguinipes makes it a useful model for the study of life history evolution across a variety of environments (Dingle et al 1990; Dingle & Mousseau 1994). This species exhibits a great deal of flexibility in its life history traits, including diapause, across its very broad geographic range.

In the northern Great Plains of North America, populations of M. sanguinipes are univoltine. Eggs are laid in the late summer and fall and embryonic development continues until diapause or cold temperatures halt development (Moore 1948; Fisher 1993). Once in diapause, morphological development does not readily resume until the embryos have experienced a period of chilling. Typically, in populations of M. sanguinipes from the northern Great Plains, diapause begins when the embryo has completed about 80% of its development (Salt 1949; Moore 1948). In alpine and high latitude environments, eggs of many species of grasshopper may require two seasons to hatch, not because of an extended diapause, but simply because of the short growing season (Kreasky 1960; Fielding, *personal observation*). In these environments, embryonic development is interrupted at early stages by the onset of winter. The following summer, development resumes until the embryo enters diapause. The eggs are then ready to hatch after overwintering a second time.

In areas with longer growing seasons, populations may be bivoltine. Dean (1982) showed that M. sanguinipes from Kansas produced a high proportion of non-diapausing eggs when parents were exposed to increasing photoperiods. Dingle et al. (1990) and Dingle & Mousseau (1994) reported that incidence of non-diapausing eggs differed among populations of M. sanguinipes in California, varying from 0 to 100% along an altitudinal gradient, with highest and lowest elevation sites exhibiting the greatest proportion of diapausing eggs. Furthermore, the age at which the embryos entered diapause varied among these populations. Dingle & Mousseau (1994) suggested that individuals from short season environments gained a fitness advantage by diapausing at later ages, enabling earlier hatching in the spring and maximizing the time available for post-embryonic growth and reproduction.

Working with Melanoplus bivittatus, Church & Salt (1952) describe diapause as facultative, stating that diapause may be averted in individuals that would otherwise diapause if the embryos were subjected to cool temperatures before entering diapause. Data reported by Fisher (1993) suggested that facultative diapause may be common in M. sanguinipes, being found in populations from Montana and Arizona. In Canada, entomologists assumed facultative diapause in the development of models predicting the timing of egg hatch based on the amount of embryonic development that took place the previous year (Gage et al. 1976).

The studies cited above suggest that embryonic diapause in M. sanguinipes may be either facultative through maternal effects, perhaps mediated by the photoperiod experienced by the parent, facultative by exposure of prediapause embryos to low temperatures, or obligate, wherein diapause cannot be averted by the environment experienced by the parent or the embryo. Also, it appears that the age at which diapause occurs may differ within and between populations. These traits may affect fitness by determining voltinism, time of hatching, and length of time available for growth and reproduction. Consideration of these traits suggests that trade-offs may be important in determining optimal diapause strategies. For instance, diapause at late stage of development minimizes the amount of development that must take place after overwintering and thus ensures an early hatching date. But, when diapause is obligate and at a late stage of development, embryos may not reach diapause age in the first year and so require two years to hatch. When embryos diapause at an early stage of development or are able to avert diapause, a greater proportion of the population will be univoltine, rather than semivoltine, but at the cost of later hatching the following year. In this case, they will have less time available for reproduction and their eggs will be produced later in the season and overwinter at an even earlier stage of development.

Analyses of the optimal timing of diapause induction have contributed to the development of the theory of evolution of life history traits in unpredictable environments (Cohen 1970; Taylor 1986a, b; Bradshaw et al. 1998; McNamara 1994; Bradford & Roff 1997). These previous studies have made some simplifying assumptions regarding diapause traits that may reduce the heuristic value of these models under some circumstances. For example, most models have assumed that the age at which diapause occurs does not vary, and that once committed to diapause it is irreversible (Taylor 1986a, Taylor & Spalding 1988, 1989). These models also assumed that if the organism is not in diapause it cannot survive winter conditions (Taylor & Spalding 1989; McNamara 1994). The model of Bradford & Roff (1997), describing maternal induction of embryonic diapause in crickets, implicitly assumed that all eggs reach diapause age in the fall. Furthermore, most analyses have been for bi- or multi-voltine populations and have ignored the role of diapause in univoltine or semivoltine populations (Taylor 1986a, b; McNamara 1994; Bradford & Roff 1997). Although these assumptions may have been justified by the particular objectives for which the model was developed, many organisms exhibit much more flexibility in their life cycle (Danks 1991).

The analysis of these more flexible strategies is problematic because the fitness effects are fully realized only in subsequent generations. For instance, diapause age and the ability to avert diapause will affect hatching dates of offspring and their reproductive success. Furthermore, in the second generation, a greater proportion of offspring of the late hatching individuals will be produced relatively late in the season, allowing for less development before the end of the season. Because of the complications involved when fitness effects are delayed (Ariew and Lewontin 2004), numerical methods were used to evaluate these different diapause strategies. In this paper, diapause characteristics are described for populations of M. sanguinipes from two very different environments, Alaska and Idaho, and a multi-generational simulation model is used to examine how assumptions regarding facultative diapause and the age at which diapause occurs may alter optimal diapause strategies over a wide range of season lengths.

## Materials and Methods

Laboratory colonies of M. sanguinipes were initiated with at least 200 individuals collected near Lewiston, Idaho and Delta Junction, Alaska as 4th and 5th instars in mid-July of 2003. Grasshoppers were maintained on romaine lettuce and wheat bran, at room temperature with incandescent lights (16 h photoperiod) suspended above the cages to allow the grasshoppers to self-regulate their internal temperatures. Trays of moist sand were provided for oviposition. Offspring from the field-collected grasshoppers were reared under the same conditions described above, and all experiments were conducted on F2 generation eggs. Climatic data from both areas were obtained from the Western Regional Climate Center (WRCC 2004). The weather station in Lewiston, Idaho was located at 46.38° North latitude, and 117.02° West longitude. Grasshoppers were collected about 5 km SW of the weather station at an elevation of about 450 m. The weather station near Big Delta, Alaska, was located at 64.00° North latitude, and 145.73° West longitude. Grasshoppers were collected about 20 km SE of the weather station at an elevation of about 400 m.

**Figure 1.**
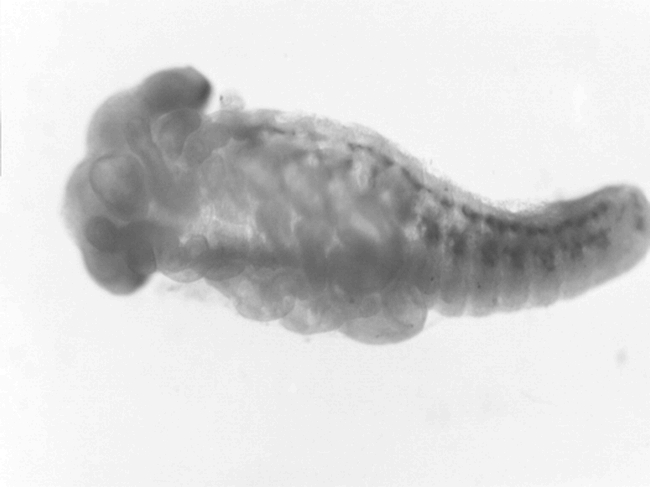
Embryo of Melanoplus sanguinipes after 12 days at 22° C, or about 40% relative development, pre-katatrepsis.

**Figure 2.**
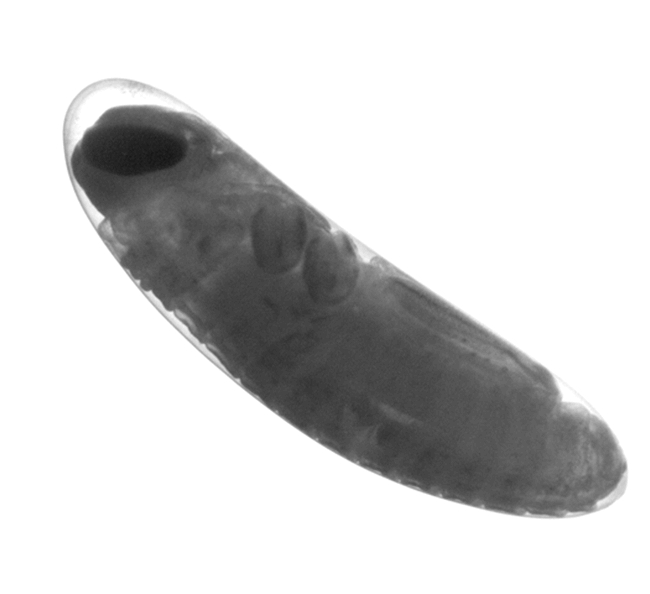
Embryo of Melanoplus sanguinipes after 32 days at 22° C, at about 80% relative development, the stage at which diapause occurs.

Oviposition trays were sifted daily to obtain egg pods of known age. Egg pods were stored in moistened vermiculite in plastic cups (50 ml) with perforated lids. Eggs were held at 22° C (± 1°) until transfer to 5° C at 7, 12, 17, 22, 27, 32, 37, and 42 days after oviposition. If development were direct, without diapause intervening, eggs should hatch in about 35 days. At least 10 pods from each population and age category were used. Two to five eggs from each pod were examined to determine the embryos' stage of development at the time of transfer to 5° C (Figs. 1 and 2). Additional eggs were also examined and categorized according to stage of development from pods not used for hatching. Embryos were categorized into 10 intervals, roughly corresponding to percentage development, using the classification scheme described by Moore (1948) where stage ten is complete embryogenesis (hatching). After 90 to 100 days, the eggs were removed from the cool conditions and maintained at room temperature and inspected daily for hatching. The number of eggs hatching was recorded daily. After 30 days at room temperature, the eggs were chilled a second time at 5° C for another 90 to 100 days, after which they were again returned to room temperature where hatching was recorded daily. The total number of eggs to hatch after the first and second periods of chilling was used as the denominator in calculating the proportion hatching after the first period chilling only. Examination of unhatched eggs after the second cold period did not reveal any remaining viable eggs (< 10% of all eggs in all treatments).

## Simulations

Optimal diapause strategies over a range of season-lengths and with different physiological constraints were examined by simulated evolution of three traits controlling diapause expression; 1) maternal control of diapause potential (as a function of day of year), 2) the age at which the embryo enters diapause, and 3) the age at which diapause could be averted by chilling (facultative diapause). Each individual in the simulations possessed four parameters that controlled diapause expression. This model was not intended to simulate biological genetic mechanisms, but was a heuristic method of finding optimal solutions akin to a genetic algorithm with stochastic sampling (Mitchell 1996). The probability that an offspring will be a potential diapauser or non-diapauser was determined by 2 parameters of the parent, N and C, which were parameters of a sigmoid equation:
				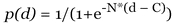 where *p(d)* is probability of diapause for offspring born on day d, N controls the abruptness of the switch from non-diapause to diapause, and C determines the timing of the switch, i.e., the day at which *p(d)* = 0.5. The age at which diapause began was determined by one parameter, *a*_d_, with possible values ranging from 12 to 30 days (corresponding to 33 and 83 % development, respectively). The age at which facultative diapause could be averted by overwintering was determined by a parameter, *a*f, with values ranging from 1 to 36 days, with any value greater than >= *a*_d_ (diapause age) corresponding to obligate diapause. Individuals were modeled as parthenogenetic females. After reaching reproductive age, individuals produced offspring at the rate of one per day. To examine the effects of a pattern of reproduction that may be more biologically realistic, simulations were also conducted with individuals producing 16 offspring every 16 days (see Appendix).

All offspring inherited the parent's traits. Parameters of individuals able to produce the most offspring gradually came to dominate the population. To avoid having the population converge to sub-optimal solutions, mutation of the inherited parameters occurred with probability of 0.001. If mutation occurred, a normally distributed value, centered on a mean of the current parameter's value and standard deviation of 20% of the mean, was substituted for the parent's parameter.

The simulations had a time step of one day. Simulations were conducted with mean season-lengths from 60 to 200 days, in increments of 10 days. Within a simulation, season-lengths were randomly drawn from a Gaussian distribution with standard deviation equal to 10% of the mean (Press et al. 1992). For comparison purposes, simulations were also conducted with constant season-lengths (see Appendix). Developmental times were not allowed to vary. Eggs required 36 days to hatch and 50 days were required from egg hatch to first reproduction. Eggs in diapause did not age and could be released from diapause only by overwintering. Only individuals in the egg stage survived between seasons, but diapause status had no relation to egg survival.

The simulations were initiated with a population of 10,000 individuals with parameters uniformly randomly distributed within their range of values. Each simulation was run for 200 generations, although the parameter frequencies generally stabilized with 50 generations. Constant daily mortality rates were 0.03 for immatures and reproductives and 0.01 for eggs (diapausing as well as those not in diapause). Overwintering eggs were subject to non-selective, density-dependent mortality to regulate the population.

Different diapause strategies were evaluated under three sets of diapause characteristics representing different levels of flexibility in life cycles. Parameters determining maternal control of diapause potential (*C* and *N*) were allowed to vary in all simulations. In the first set of simulations, age at which diapause began, *a*_d_, was fixed at 30 days for all individuals with no possibility of averting diapause (obligate diapause). In the next set of simulations, *a*_d_ was allowed to vary, but again with no possibility of averting diapause. For the third set, all parameters were allowed to vary. For each set of options, three simulations were run at each mean season-length.

## Results

Long term climate data show that the mean number of consecutive freeze-free days (0° C) was 186 in Lewiston, Idaho, with 80% of years (1949 through 2003) having between 151 and 215 freeze-free days. In Big Delta, Alaska, mean season length was 105 days, with 80% of years (1937 through 2003) having between 85 and 125 freeze-free days. Mean annual temperatures were 11.3 and −2.2 °C for Lewiston, Idaho, and Big Delta, Alaska, respectively.

**Figure 3.**
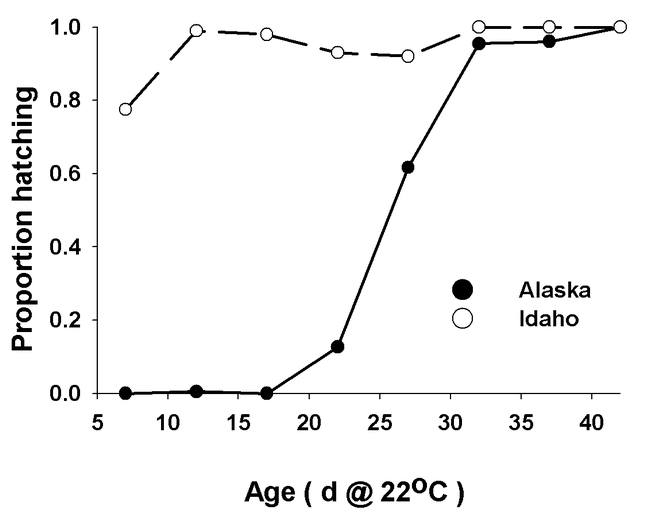
Proportion of eggs from two populations of Melanoplus sanguinipes that hatched after exposure to 5° C for 90 - 100 days following incubation at 22° C for different times. Closed circles, Alaska; open circles, Idaho. N > 200 at each point.

Embryos of the Alaskan population developed somewhat more slowly than the Idaho population (Table 1) at the temperature at which the eggs were maintained in this experiment. The Idaho embryos were mostly at stage VI (katatrepsis) at 17 days, whereas the majority of the Alaskan embryos did not reach this stage until 22 days. Both populations diapaused at about 80% of total embryonic development (Fig. 2), with nearly all embryos at stage VIII (Moore 1948) by 32 days. The populations differed in their response to pre-diapause chilling (Fig. 3). Diapause was averted in more than 75% of the Idaho eggs when exposed to cool temperatures at age 7 days, and over 90% hatched when exposed to cool temperatures at ages of 12 days (Fig. 3) and older. In contrast, diapause appeared to be obligate in the Alaska population, or, at least, diapause could not be averted until after katatrepsis (Fig. 3). The difference in developmental rates between the two populations was not great enough to fully account for the differences in ages at which cold treatment averted or terminated diapause. All viable eggs hatched after the second period of chilling.

**Table 1.**
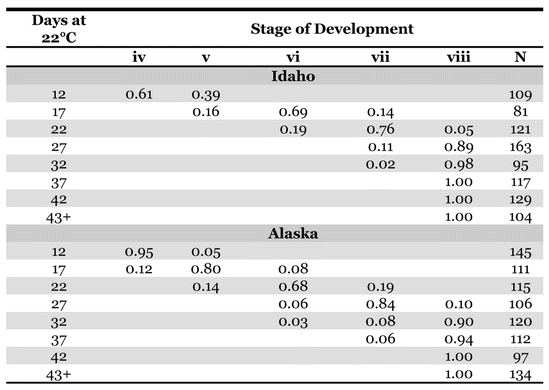
Proportion of the population within stages of embryonic development (Moore 1948) with increasing incubation time.

## Simulation results

**Figure 4.**
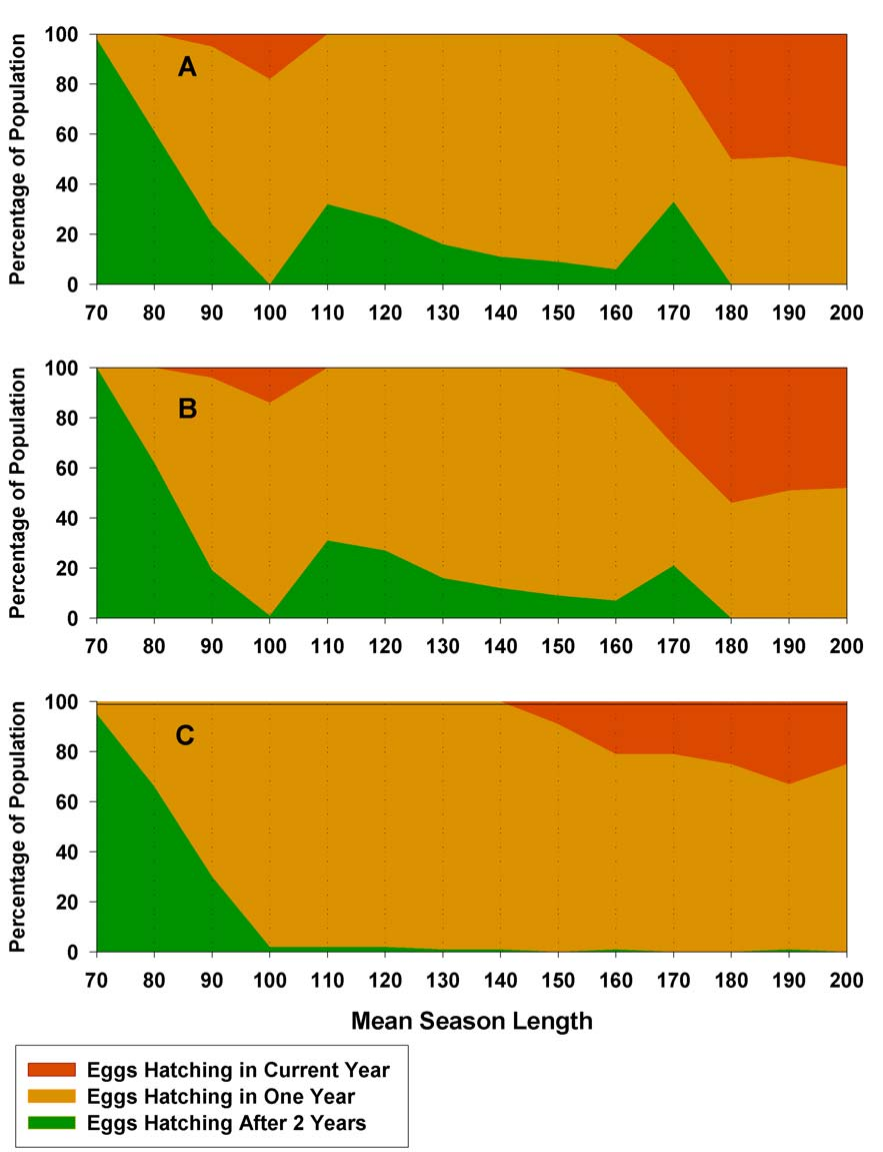
Patterns of voltinism with increasing season-lengths under different assumptions of embryonic diapause in Melanoplus sanguinipes. Within a simulation, season-lengths varied randomly, normally distributed with standard deviation equal to 10% of mean season-length. In all cases, diapause potential is maternally determined. A) Diapause is obligate in all eggs with diapause potential, and diapause age is fixed at 30 d, B) Diapause is obligate in all eggs with diapause potential, but diapause age was allowed to vary, C) Diapause could be averted by overwintering pre-diapause embryos.

The different models of diapause development generated different patterns of voltinism and different optimal diapause strategies, particularly when mean season-length was near some multiple of generation time. Minimum generation time with daily reproduction was about 90+ days: 36 days for embryogenesis, 50 days to first reproduction, plus 6 to 10 days to produce enough offspring for replacement. When the growing season was less than this, a semivoltine life cycle predominated (Fig. 4). Populations went extinct at mean season-lengths less than 70 days. At mean season-lengths of 180 days and longer, a substantial second generation was possible.

**Figure 5.**
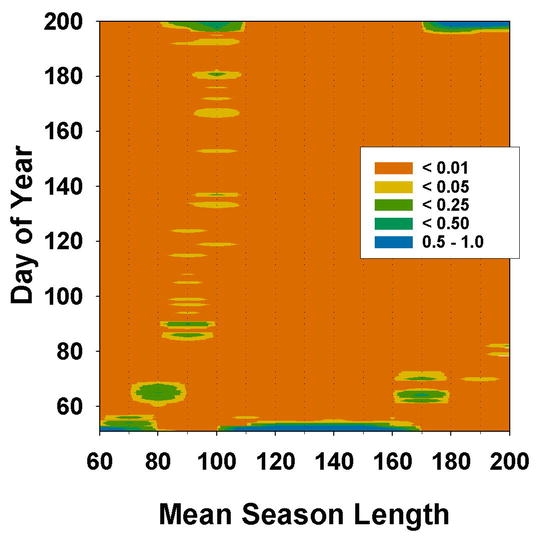
Frequency distribution of median date of maternally determined switch from non-diapausing eggs to eggs with diapause potential. Contours indicate proportion of the population after 200 generations. Diapause was obligate in those eggs with diapause potential and diapause age fixed at 30 days. If diapause switch date was greater than season length, all eggs were non-diapausing.

In simulations where diapause was obligate for those embryos with maternally induced diapause potential (*a*_f_ >= *a*_d_), some surprising patterns emerged (Fig. 4A). At very short seasons (70 days), populations were semivoltine and all eggs diapaused, as expected. But, as season-length increased to the point where a univoltine life cycle became possible (80 to 100 days), the predominate strategy was that of direct development to hatching, i.e., no maternally induced diapause potential. At 80 and 90 days mean season-lengths, females produced mostly non-diapause eggs until late in the season (Fig. 5). At 100 days, females produced exclusively non-diapausing eggs. At these transitional season-lengths, a univoltine life cycle was possible, at least for eggs laid early in the season, but the seasons were not long enough for the majority eggs to reach diapause stage in their first year. If the embryos were to diapause, it would occur during the following season, thereby forcing a semivoltine life cycle. By not diapausing, all eggs hatched the following year and there was no semivoltinism (Fig. 4A). There were costs associated with this strategy. Because many of the eggs hatched later than if they were in late-stage diapause, their reproductive time was abbreviated. Also, a small proportion of these non-diapausing eggs hatched in the current year (Fig. 4A), but too late to grow to adult stage. At season-lengths greater than 100 days, a univoltine life cycle was possible for diapausing eggs. Therefore, females produced only diapausing eggs at season-lengths from 110 to 160 days (Fig. 5). Nevertheless, at season-lengths from 110 to 160 days, obligate diapause carried with it the cost of some proportion of the population, those eggs laid late in the season, not reaching diapause age the first year, and being constrained to a 2 yr life cycle (Fig. 4A). This pattern was repeated at season-lengths of 170 to 200 days, as a second generation became possible. At 170 days mean season-length, females began to produce some non-diapausing eggs early in the season (Fig. 5), and from 180 to 200 days females produced non-diapausing eggs exclusively for the same reasons as described above for the 100 day season-length. Again, some proportion of the non-diapausing eggs that hatched too late in the current year represented wasted reproductive effort because they did not have time to complete development before the end of the season.

**Figure 6.**
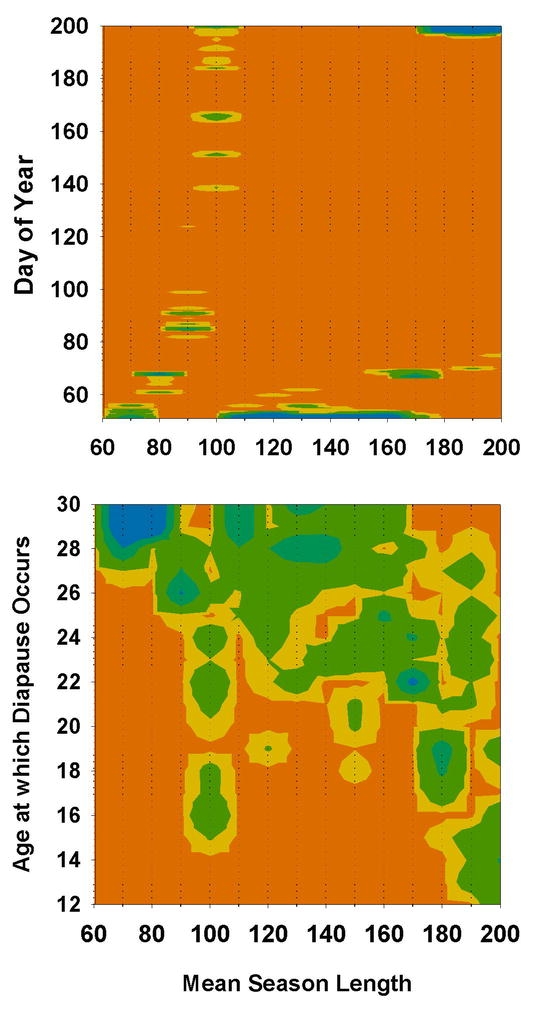
Diapause strategies when diapause age was allowed to vary. Upper panel as in Fig. 5. Lower panel: frequency distribution of age at which diapause occurred. Contours indicate proportion of the population after 200 generations.

Allowing diapause age to vary did not change these patterns substantially (Figs. 4B, 6). At season-lengths from 70 to 90 days, diapause age was restricted to late stages (Fig. 6). Because at these short seasons a two-year life cycle predominates, embryos have the entire season to reach a late stage of development, which allows for an early hatch after the second winter. At 100 days, there was no selection pressure on diapause age because, through maternal effects, no embryos diapaused (C > 120 days, Fig. 6). At season lengths from 110 to 160 days, the range of diapause ages in the population broadened, but never less than 19 days (Fig. 6). Within this range of season lengths, earlier diapause ages would be of no advantage because any increase in the number of eggs reaching diapause age before winter, due to earlier diapause age, would be offset by later hatching and later dates of first reproduction. Also, embryos entering diapause at an early age may not be able take advantage of the full time available for development during the first year. Again, at season-lengths greater than 170 days, there was no selection on diapause age because no eggs diapaused (Fig. 6). Bivoltinsim at mean season-lengths greater than 170 days, under assumptions of obligate diapause (Fig. 4A, B), is inflated by those eggs hatching but not completing development before the end of the season.

**Figure 7.**
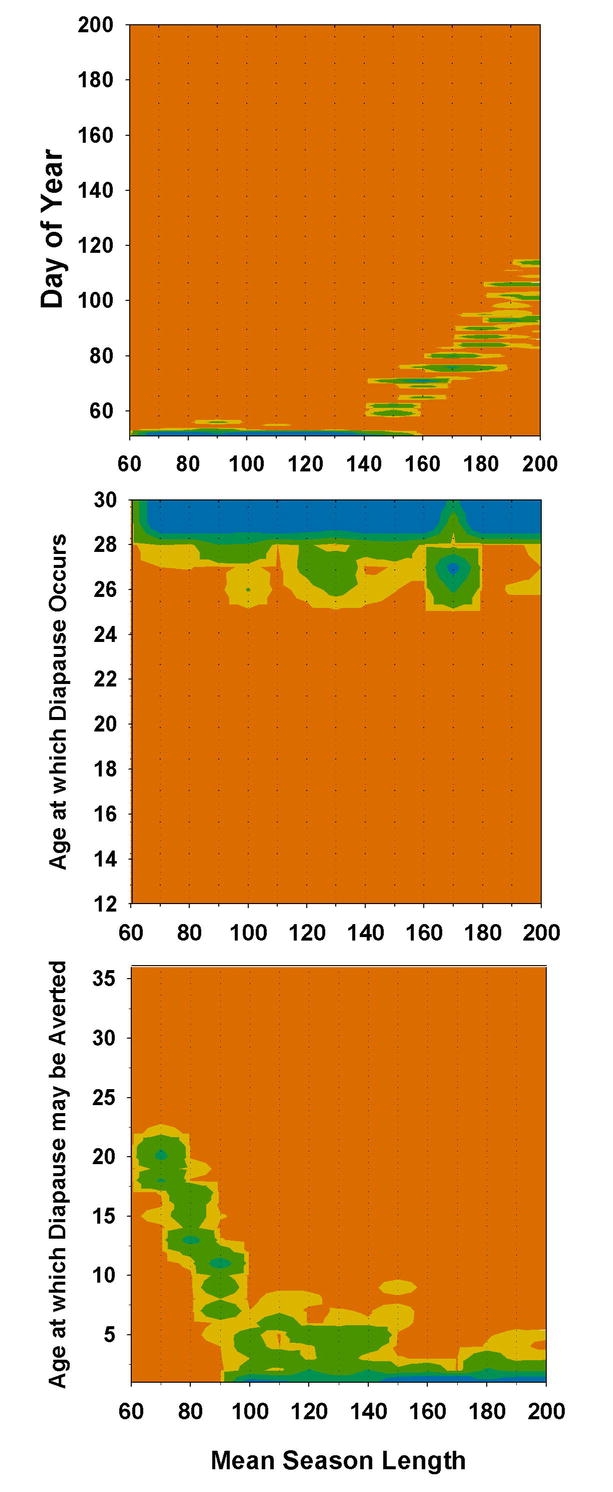
Diapause strategies when diapause could be averted by overwintering. Upper and middle panels as in Figs. 5 and 6. Lower panel: Frequency distribution of the minimum age at which embryo was able to avert diapause. Contours indicate proportion of the population after 200 generations.

Facultative diapause, i.e., avoidance of diapause by overwintering pre-diapause embryos, allowed individuals to circumvent the trade-offs inherent in the simple diapause versus no-diapause strategies of obligate diapausers. Eggs that did not reach diapause stage could still hatch the following year, without the risk of hatching in the current year too late to complete development. Diapause aversion eliminated much of the inefficiencies associated with obligate diapause, such as semi-voltinism at season-lengths from 110 to 160 days (Fig. 4). At mean season-lengths greater than 90 days, most individuals were able to avert diapause at very early ages (Fig. 7). At season-lengths of 70 days, populations were mostly semi-voltine, but a small proportion of eggs produced early in the season were able to hatch the following year (Fig. 4). The minimum age at which diapause could be averted was limited to about 17 to 23 days (Fig. 7). If they averted diapause at an earlier age, they would hatch later the following year, with increased probability of not being able to complete development at these short seasons. If they were not able to avert diapause until older than 23 days, there was little chance of them reaching that age in the current year, and thus would enter diapause anyway during the following year and so take 2 years to hatch. When age at onset of diapause and age at which diapause could be averted were allowed to vary simultaneously, diapause ages < than 26 days did not persist in the population (Fig. 7). With facultative diapause, dates of the switch from non-diapausing to diapausing eggs corresponded to the classical population response of progressively later dates with increasing length of season (Fig. 7). Facultative diapause allowed bivoltinism to begin about at season-lengths about 10 days shorter than with obligate diapause (Fig. 4).

Simulations with constant season-lengths and with reproduction at intervals (16 eggs every 16 days), did not greatly change the qualitative patterns described above (see Appendix).

## Discussion

By considering the evolution of three different traits simultaneously, the effect of life cycle flexibility on optimal diapause strategies was evaluated. Most, if not all, previous analyses of diapause timing have assumed that diapause age is fixed, and once committed to diapause, it cannot be averted. To my knowledge, this is the first study to systematically examine the implications of different assumptions regarding diapause development over a wide range of season lengths. The ability to avert diapause was shown to potentially influence the incidence of diapause, voltinism at certain season-lengths, the timing of diapause induction, and to increase the flexibility of population responses to variable season-lengths. The age at which embryonic diapause may occur apparently varies among some populations of M. sanguinipes (Dingle & Mousseau 1994), but this trait did not have a great influence on life histories in the simulations.

It is very difficult to determine the actual length of the growing season, in terms of generation time, as experienced by grasshoppers. Many factors in addition to temperature determine developmental time of insects: dry soil may delay embryonic development (Mukerji and Gage 1978, Colvin 1996), placement of egg pods may affect their thermal environment (Kemp & Sanchez 1987), food quality may affect development of nymphs and reduce fecundity of adults (Hewitt 1985; Awmack & Leather 2002; Danner & Joern 2004), and developmental rates may differ between populations (Dingle et al 1990; Dingle & Mousseau 1994; Fielding 2004). Due to the difficulty in determining length of the growing season as experienced by the grasshoppers, precise quantitative predictions with respect to optimal diapause strategies in any particular location are correspondingly difficult to make. It may be possible though, to match model predictions with empirical data in terms of qualitative patterns.

**Appendix Figure 3.**
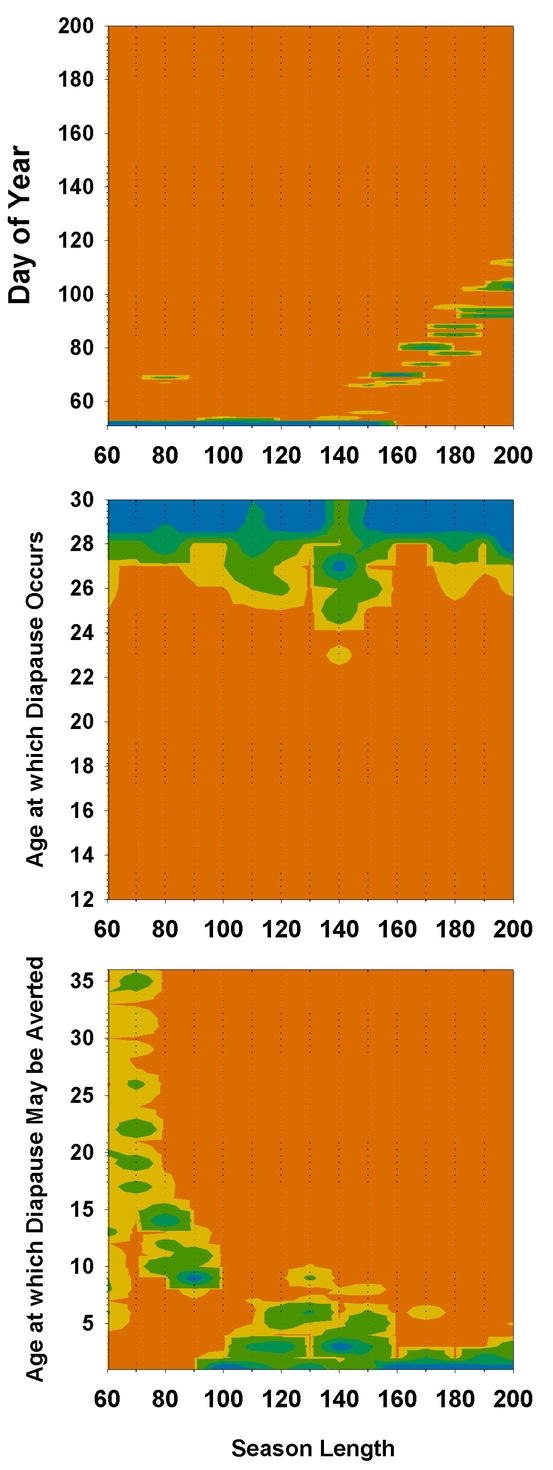
Results of simulations with constant season-lengths. Diapause strategies when diapause could be averted by overwintering. Upper and middle panels as in Figs. 1 and 2. Lower panel: Frequency distribution of the minimum age at which embryo was able to avert diapause. Contours indicate proportion of the population after 200 generations. Season-length varied with SD = 10% of the mean.

The apparent inability of Alaskan M. sanguinipes to avert diapause was not reproduced in the simulations. Even at the shortest season simulated, if diapause aversion was allowed, strategies emerged that averted diapause several days before the diapause age (Fig. 7). At very short constant season-lengths, eggs do not reach an age where averting diapause would be an advantage, so there was no selection pressure against the ability to avert diapause (Appendix Fig. 3). Under the more realistic assumption of stochastic season-lengths, there is some advantage to averting diapause, even at the shortest seasons at which populations could persist. With season-lengths of mean = 70 days with SD = 7 days, some years would be long enough for some portion of the population to achieve a univoltine life cycle, but many years would not be long enough. Under these circumstances, the minimum age at which diapause could be averted was limited to a narrow range of ages between 17 and 23 days (Fig. 7). Only eggs laid early in the season were able to reach this age in most years. If these embryos averted diapause and the following year was unusually short, there was a risk of not being able to complete development, but if embryos waited to avert diapause at later ages, they risked a missed opportunity for a univoltine life cycle in years with longer seasons. In any case, females were able to hedge their bets because a large proportion of reproduction occurred too late in the season for the embryos to be old enough to avert diapause and so required two years to hatch anyway, but were also ensured of surviving unusually short seasons.

There are several possibilities that could explain the inability of Alaskan M. sanguinipes to avert diapause. If the ability to avert diapause is an all-or-nothing trait, i.e., either the embryo can avert diapause regardless of age or not at all, then the observed response of the Alaskan eggs is easily explained. Another possibility may be that some small percentage of Alaskan embryos averted diapause, but at a stage of development near the diapause stage (perhaps not all of the eggs aged 22 and 27 days that hatched after the first cold treatment were in diapause). Empirical studies are planned to examine these possibilities. It may be that there are other factors besides season-length selecting for obligate diapause. Populations in Alaska are semivoltine, and cohorts from odd-numbered years are much reduced compared to those of even-numbered years. Alternating population abundances is not unusual in insects with 2-year life cycles (Heliovaara et al. 1994). Density-dependent factors are necessary to enforce this alternate year pattern. The factors that suppress populations of M. sanguinipes in alternate years, whatever they may be, may also select against a one-year life cycle, regardless of season-length. Studies are underway to examine the role of parasitoids and pathogens in the dynamics of subarctic populations of acridids.

The traits of the Idaho population conformed to predictions from the simulations. Diapausing at a late stage of development, but able to avert diapause early, is an optimal strategy in most environments. Other published data suggests that it is a taxonomically and geographically widespread phenomenon in acridids (Church & Salt 1952; Gage et al. 1976; Fisher 1993).

Dingle and Mousseau (1994) described an altitudinal gradient of diapause incidence in M. sanguinipes wherein populations at the highest and lowest altitudes (and most likely, shortest and longest seasons) had the highest incidence of diapausing eggs, and at intermediate altitudes non-diapausing eggs were common. Although they did not test for facultative diapause, this gradient could possibly be produced by populations with an obligate diapause if the intermediate altitudes spanned a transition in voltinism (Figs. 4 and 5). In an earlier paper, Dingle et al. (1990) state that all these populations were univoltine, but did not present any evidence with respect to voltinism.

There is general interest in understanding and predicting the response of various insect species to climate change (Bradshaw & Holzapfel 2001; Post et al. 2001). Bradshaw et al. (2000) proposed that photoperiodically timed seasonal development is the primary adaptive response of species to climate. Flexible diapause strategies can increase an organism's ability to adapt to changing climates. For example, in simulations described here, facultative diapause allowed bivoltinism to appear at relatively shorter seasons than populations with obligate diapause. A full understanding of an insect's diapause traits are therefore essential to understand population responses to changing climates.

An understanding of diapause is also important for pest management planning and for parameterization of simulation models (Pickford 1966; Gage et al. 1976; Fisher et al 1996, 1999). In Montana, models of post-diapause hatching (Kemp & Sanchez 1987) assumed that all eggs were in the diapause stage by onset of winter and were at the same stage of development in the spring. But in Saskatchewan, Moore (1948) reported wide variation in embryonic development in late fall, with very few eggs having entered diapause. Models of grasshopper phenology by Gage et al. (1976) base their estimates of time of hatch on the amount of development in the previous year, ignoring the possibility that some of these eggs may enter diapause and not hatch until the second spring. If embryos are in various stages of development in the spring, and have a facultative diapause so that they all hatch, then hatching will occur over a wider time frame. In either case, such assumptions need to be tested explicitly for more reliable predictions of phenology.

The results presented here also suggest the possibility that some portion of the population, primarily those eggs produced late in the season, may not hatch the first year after oviposition, even in areas with relatively long seasons. This would be more likely if diapause could not be averted at a very early age. Whether a significant proportion of these eggs can survive that long is an open question, but one that may worth investigation. In seasons with a late flush of vegetative growth, or in unusually short seasons, a substantial proportion of eggs may not have developed enough to avert diapause and not hatch the following year. Two years hence, these eggs may hatch along with the one-year-old eggs of the previous year, to produce a greater than expected population. Such phenomena may also reduce the multi-year benefits of controlling grasshopper populations. Across much of the geographic range of M. sanguinipes, where univoltine life cycles predominate, most adults may die well before the end of the season and most oviposition may occur early enough such that nearly all eggs diapause, in which case facultative or obligate diapause may not be relevant to population dynamics. Also, in many regions, eggs may not survive two years in ground due to desiccation or predation. Obviously, these conditions will vary greatly between years.

Further examination of the diapause traits of different populations of M. sanguinipes, and other species, is needed to validate and refine the simulations presented here. Certain assumptions in this simulation may also have influenced the results. For example, perfect inheritance was a feature of this model. Taylor & Spalding (1989) showed that with perfect inheritance optimal switching times from non-diapausing to diapausing were more conservative, that is earlier, than when inheritance of this trait was < 1.0. Also, more research is needed to determine whether the phenotypic expression of traits, such as age at which diapause begins, is plastic or is determined purely by genotype. If the environment experienced by individuals affects these traits, life cycles may be even more flexible than were modeled here. The results of these simulations suggest that assumptions regarding diapause traits can affect the results of life history analyses, predictions of population responses to climate change, and applications of phenological models. These assumptions regarding facultative diapause and age at which diapause occurs need to be made explicit and examined so that the relevance of such analyses to a particular population can be assessed.

## Figures and Tables

**Appendix Figure 1.**
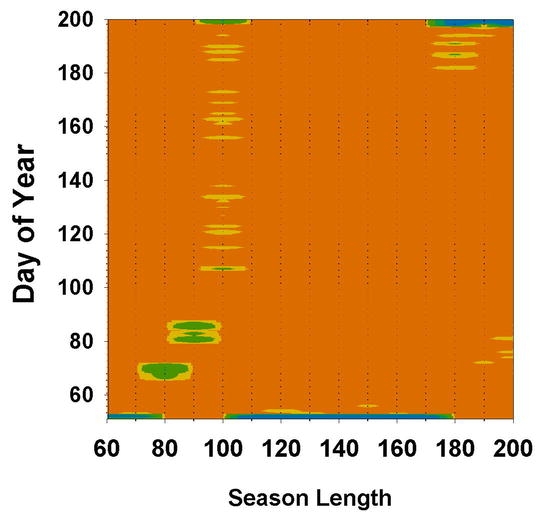
Results of simulations with constant season-lengths. Frequency distribution of median date of switch from eggs without maternally determined diapause potential to eggs with diapause potential. Contours indicate proportion of the population after 200 generations. Diapause was obligate in those eggs with diapause potential and diapause age fixed at 30 days. If diapause switch date was greater than season length, all eggs were non-diapausing. Diapause strategies when diapause could be averted by overwintering. Upper and middle panels as in Figs. 5 and 6. Lower panel: Frequency distribution of the minimum age at which embryo was able to avert diapause. Contours indicate proportion of the population after 200 generations.

**Appendix Figure 2.**
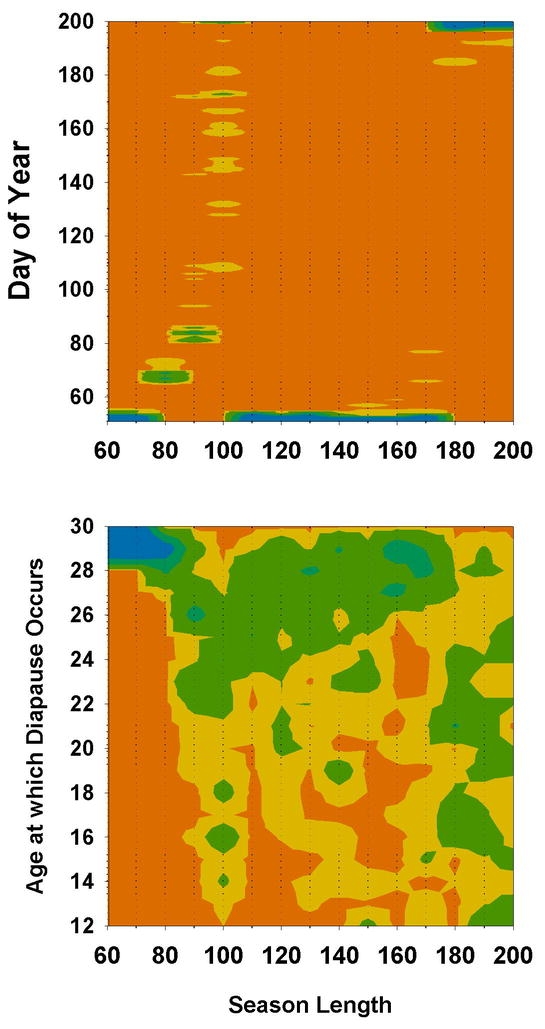
Results of simulations with constant season-lengths. Diapause strategies when diapause age was allowed to vary. Upper panel as in Appendix Fig. 1. Lower panel: frequency distribution of age at which diapause occurred. Contours indicate proportion of the population after 200 generations.

**Appendix Figure 4.**
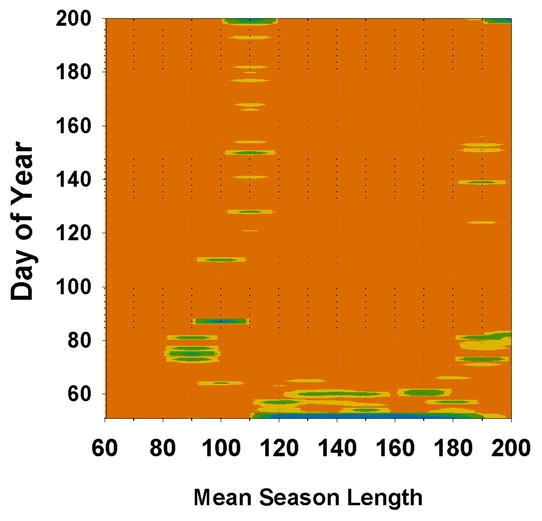
Results of simulations with constant season-lengths. Diapause strategies when diapause age was allowed to vary. Upper panel as in Appendix Fig. 1. Lower panel: frequency distribution of age at which diapause occurred. Contours indicate proportion of the population after 200 generations.

**Appendix Figure 5.**
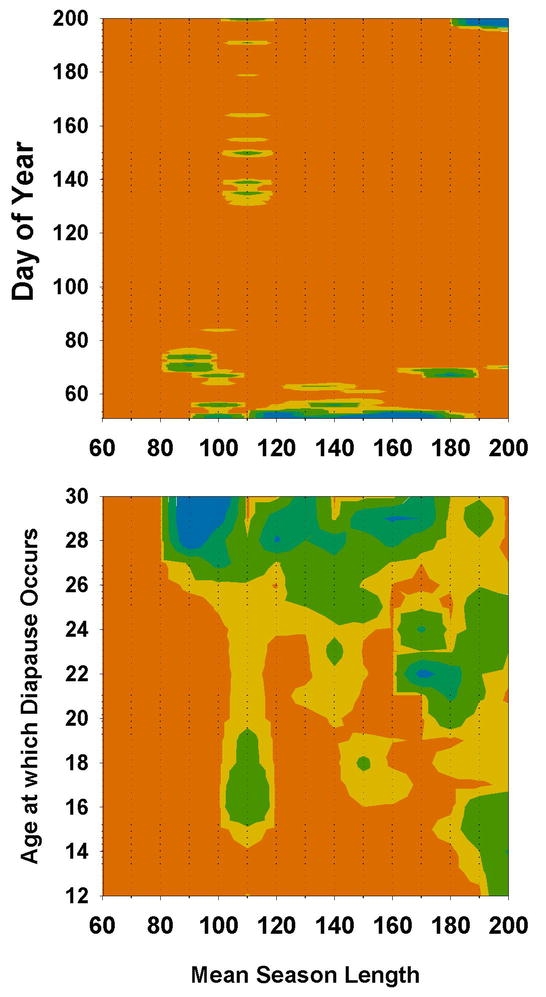
Results of simulations with reproduction at intervals (16 eggs every 16 days). Diapause strategies when diapause age was allowed to vary. Upper panel as in Fig. 5. Lower panel: frequency distribution of age at which diapause occurred. Contours indicate proportion of the population after 200 generations.

**Appendix Figure 6.**
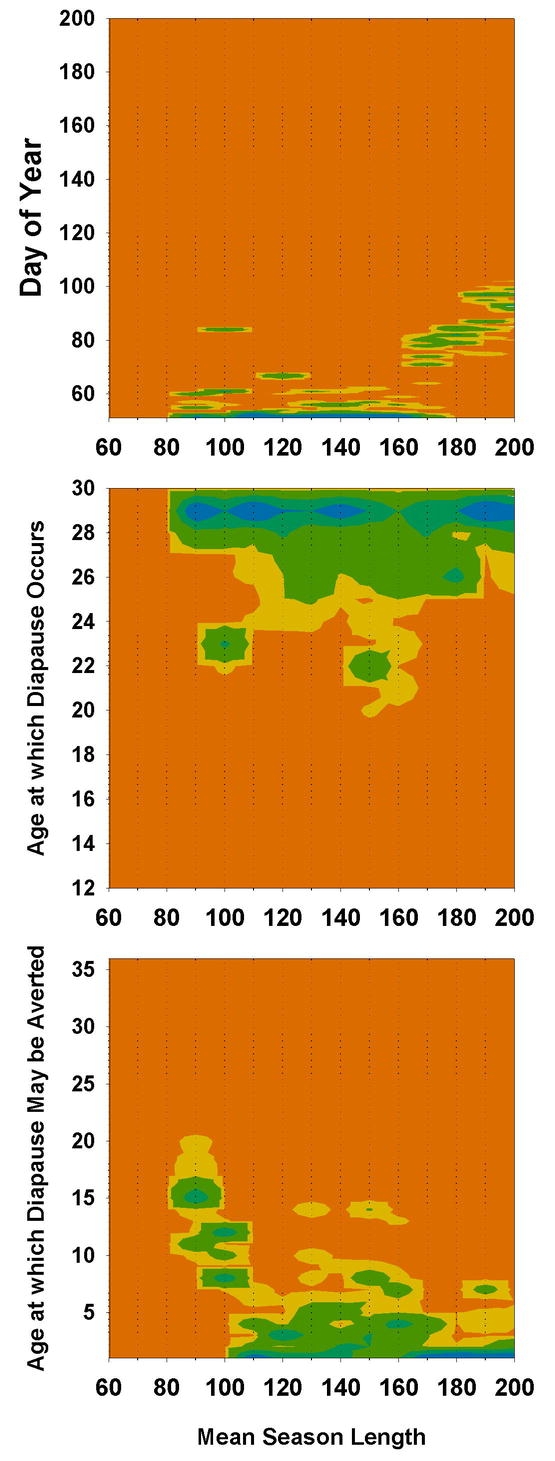
Results of simulations with reproduction at intervals (16 eggs every 16 days). Diapause strategies when diapause could be averted by overwintering. Upper and middle panels as in Figs. 5 and 6. Lower panel: Frequency distribution of the minimum age at which embryo was able to avert diapause. Contours indicate proportion of the population after 200 generations. Season-length varied with SD = 10% of the mean.
